# Supplementary material for: The Impact of IL-6 and IL-10 Gene Polymorphisms in Diffuse Large B-Cell Lymphoma Risk and Overall Survival in an Arab Population: A Case-Control Study
Source: Cancers (Basel). 2020 Feb 7;12(2):382. doi: 10.3390/cancers12020382 (PMC7072608; doi:10.3390/cancers12020382)
Supplement: Supplementary file 1 [file cancers-12-00382-s001.zip › Table S1.pdf]

Table S1. The Hardy Weinberg equilibrium (HWE) p-values for the cases and the controls.

**rs1800795**	**HWE* *p*-value**	**HWE* *p*-value**
Allele G	1	0.78
Allele C		
Genotype G/G	1	0.78
Genotype C/G		
Genotype C/C		
Genotype C/G-C/C		
Genotype G/G-C/G		
Genotype G/G-C/C		
**rs1800796**		
Allele G	0.15	0.85
Allele C		
Genotype G/G	0.15	0.85
Genotype G/C		
Genotype C/C		
Genotype G/C-C/C		
Genotype G/G-G/C		
Genotype G/G-C/C		
**rs1800797**		
Allele G	1	1
Allele A		
Genotype G/G	1	1
Genotype A/G		
Genotype A/A		
Genotype A/G-A/A		
Genotype G/G-A/G		
Genotype G/G-A/A		
**rs1800871**		
Allele G	0.37	0.55
Allele A		
Genotype G/G	0.37	0.55
Genotype G/A		
Genotype A/A		
Genotype G/A-A/A		
Genotype G/G-G/A		
Genotype G/G-A/A		
**rs1800872**		
Allele G	0.4	0.08
Allele T		

Genotype G/G<br>Genotype G/T<br>Genotype T/T<br>Genotype G/T-T/T<br>Genotype G/G-G/T<br>Genotype G/G-T/T	0.4	0.08
**rs1800890**		
Allele A<br>Allele T	1	0.73
Genotype A/A<br>Genotype A/T<br>Genotype T/T<br>Genotype A/T-T/T<br>Genotype A/A-A/T<br>Genotype A/A-T/T	1	0.73
**rs1800896**		
Allele T<br>Allele C	0.57	0.69
Genotype T/T<br>Genotype C/T<br>Genotype C/C<br>Genotype C/T-C/C<br>Genotype T/T-C/T<br>Genotype T/T-C/C	0.57	0.69

*HWE: Hardy-Weinberg equilibrium.
