# Supplementary material for: The Impact of IL-6 and IL-10 Gene Polymorphisms in Diffuse Large B-Cell Lymphoma Risk and Overall Survival in an Arab Population: A Case-Control Study
Source: Cancers (Basel). 2020 Feb 7;12(2):382. doi: 10.3390/cancers12020382 (PMC7072608; doi:10.3390/cancers12020382)
Supplement: Supplementary file 1 [file cancers-12-00382-s001.zip › Table S2.pdf]

Table S2. The Frequency of allele and genotype for seven cytokines related SNPs in DLBCL patients and controls (Case / Control).

SNP ID			
**rs1800795**	**Cases N(%)**	**Controls N(%)**	***p*-value**
Allele G	200 (85) ^	390 (86) ^	0.878
Allele C	34 (15) ^	64 (14) ^	
Genotype G/G	85 (72.7)	168 (74)	0.936**
Genotype C/G	30 (25.6)	54 (23.8)	
Genotype C/C	2 (1.7)	5 (2.2)	
**rs1800796**			
Allele G	230 (93) ^	439 (92) ^	0.804
Allele C	18 (7) ^	37 (8) ^	
Genotype G/G	106 (85.5)	202 (84.9)	1**
Genotype G/C	18 (14.5)	35 (14.7)	
Genotype C/C	0 (0)	1 (0.4)	
**rs1800797**			
Allele G	204 (85) ^	404 (86) ^	0.832
Allele A	36 (15) ^	68 (16) ^	
G/G	86 (71.7)	173 (73.3)	0.907**
A/G	32 (26.7)	58 (24.6)	
A/A	2 (1.7)	5 (2.1)	
**rs1800871**			
Allele G	165 (72) ^	313 (73) ^	0.761
Allele A	63 (28) ^	113 (27) ^	
Genotype G/G	59 (51.8)	117 (54.9)	0.756
Genotype G/A	47 (41.2)	79 (37.1)	
Genotype A/A	8 (7)	17 (8)	
**rs1800872**			
Allele G	169 (72) ^	342 (72) ^	0.813
Allele T	67(28) ^	130 (28) ^	
Genotype G/G	60 (50.9)	124 (52.5)	0.952
Genotype G/T	49 (41.5)	94 (39.8)	
Genotype T/T	9 (7.6)	18 (7.6)	
**rs1800890**			
Allele A	154 (70) ^	321 (73) ^	0.426
Allele T	66 (30) ^	119 (27) ^	

Genotype A/A	54 (49.1)	118 (53.6)	0.725
Genotype A/T	46 (41.8)	85 (38.6)	
Genotype T/T	10 (9.1)	17 (7.7)	
**rs1800896**			
Allele T	147 (60) ^	277 (59) ^	0.782
Allele C	99 (40) ^	195 (41) ^	
Genotype T/T	42 (34.1)	83 (35.2)	0.670
Genotype C/T	63 (51.2)	111 (47)	
Genotype C/C	18 (14.6)	42 (17.8)	

^ Allele frequency and percent. The allele frequency might be counted twice in each person.

** Fisher's Exact test
