# Supplementary material for: The Impact of IL-6 and IL-10 Gene Polymorphisms in Diffuse Large B-Cell Lymphoma Risk and Overall Survival in an Arab Population: A Case-Control Study
Source: Cancers (Basel). 2020 Feb 7;12(2):382. doi: 10.3390/cancers12020382 (PMC7072608; doi:10.3390/cancers12020382)
Supplement: Supplementary file 1 [file cancers-12-00382-s001.zip › Table S3.pdf]

Table S3. Genotype distribution of the IL-6 and IL-10 in DLBCL patients.

SNP ID	Model	Genotype	OR^ (95% CI)	*P*-value
**rs1800795 IL-6**	Codominant	G/G	1	0.89
		C/G	0.91 (0.54-1.53)	
		C/C	1.26 (0.24-6.66)	
	Dominant	G/G	1	0.79
		C/G-C/C	0.93 (0.56-1.54)	
	Recessive	G/G-C/G	1	0.76
		C/C	1.30 (0.25-6.78)	
	Overdominant	G/G-C/C	1	0.71
		C/G	0.91 (0.54-1.52)	
**rs1800796 IL-6**	Codominant	G/G	1	0.66
		G/C	1.02 (0.55-1.89)	
		C/C	NA (0.00-NA)	
	Dominant	G/G	1	0.88
		G/C-C/C	1.05 (0.57-1.94)	
	Recessive	G/G-G/C	1	0.36
		C/C	NA (0.00-NA)	
	Overdominant	G/G-C/C	1	0.96
		G/C	1.02 (0.55-1.88)	
**rs1800797 IL-6**	Codominant	G/G	1	
		A/G	0.90 (0.54-1.49)	0.88
		A/A	1.24 (0.24-6.54)	
	Dominant	G/G	1	
		A/G-A/A	0.92 (0.56-1.50)	0.74
	Recessive	G/G-A/G	1	
		A/A	1.28 (0.24-6.68)	0.77
	Overdominant	G/G-A/A	1	
		A/G	0.90 (0.54-1.48)	0.67
**rs1800871 IL-10**	Codominant	G/G	1	
		G/A	0.85 (0.53-1.37)	0.76
		A/A	1.07 (0.44-2.63)	
	Dominant	G/G	1	0.58
		G/A-A/A	0.88 (0.56-1.39)	
	Recessive	G/G-G/A	1	
		A/A	1.15 (0.48-2.75)	0.75
	Overdominant	G/G-A/A	1	
		G/A	0.84 (0.53-1.34)	0.46
**rs1800872 IL-10**	Codominant	G/G	1	
		G/T	0.93 (0.58-1.47)	0.76
		T/T	0.97 (0.41-2.28))	
	Dominant	G/G	1	
		G/T-T/T	0.93 (0.60-1.45)	0.58
	Recessive	G/G-G/T	1	
		T/T	1.00 (0.43-2.30)	NA*
	Overdominant	G/G-T/T	1	
		G/T	0.93 (0.59-1.46)	0.76

**rs1800890<br>IL-10**	Codominant	A/A<br>A/T<br>T/T	1<br>0.85 (0.52-1.37)<br>0.78 (0.33-1.81)	0.73
	Dominant	A/A<br>A/T-T/T	1<br>0.83 (0.53-1.32)	0.44
	Recessive	A/A-A/T<br>T/T	1<br>0.84 (0.37-1.90)	0.67
	Overdominant	A/A-T/T<br>A/T	1<br>0.88 (0.55-1.40)	0.58
**rs1800896<br>IL-10**	Codominant	T/T<br>C/T<br>C/C	1<br>0.89 (0.55-1.45)<br>1.18 (0.61-2.30)	0.67
	Dominant	T/T<br>C/T-C/C	1<br>0.96 (0.60-1.51)	0.85
	Recessive	T/T-C/T<br>C/C	1<br>1.26 (0.69-2.30)	0.44
	Overdominant	T/T-C/C<br>C/T	1<br>0.85 (0.55-1.31)	0.45

^: Odd Ratio

*NA: not available.
