# Supplementary material for: The Impact of IL-6 and IL-10 Gene Polymorphisms in Diffuse Large B-Cell Lymphoma Risk and Overall Survival in an Arab Population: A Case-Control Study
Source: Cancers (Basel). 2020 Feb 7;12(2):382. doi: 10.3390/cancers12020382 (PMC7072608; doi:10.3390/cancers12020382)
Supplement: Supplementary file 1 [file cancers-12-00382-s001.zip › Table S4.pdf]

Table S4: Multivariate Survival analyses using Cox Regression Model for SNP 1800797 and controlling for Age and Stage.

Model	OR^ (95% CI)	P-value	Model p-value
Codominant Genotype		0.002	0.000
A/A vs. G/G	1.85 (0.85-4.02)	0.122	
A/G vs. G/G	24.25 (4.2-139.8)	0.000	
Age in Years*		0.404	
15-19 vs. 0-14	0.58 (0.08-4.28)	0.59	
20-40 vs. 0-14	0.91 (0.40-2.0)	0.81	
41-55 vs. 0-14	0.46 (0.21-1.0)	0.05	
Ann Arbor Stage at Diagnosis		0.407	
1 vs. 0	1.97 (0.23-17.16)	0.540	
2 vs. 0	0.66 (0.32-1.40)	0.280	
3 vs. 0	1.73 (0.62-4.82)	0.297	
4 vs. 0	0.54 (0.16-1.76)	0.303	
Dominant Genotype			0.221
A/G-A/A vs. G/G	1.46 (0.72-2.99)	0.295	
Age in Years*		0.363	
15-19 vs. 0-14	0.53 (0.07-3.95)	0.537	
20-40 vs. 0-14	1.07 (0.48-2.35)	0.874	
41-55 vs. 0-14	0.46 (0.21-1.02)	0.056	
Ann Arbor Stage at Diagnosis		0.601	
1 vs. 0	1.97 (0.23-17.16)	0.540	
2 vs. 0	0.66 (0.32-1.40)	0.280	
3 vs. 0	1.73 (0.62-4.82)	0.297	
4 vs. 0	0.54 (0.16-1.76)	0.303	
Recessive Genotype			0.000
G/G-A/G vs. A/A	14.53 (2.87-73.53)	0.001	
Age in Years*		0.391	
15-19 vs. 0-14	0.49 (0.07-3.59)	0.48	
20-40 vs. 0-14	0.91 (0.4-2.05)	0.814	
41-55 vs. 0-14	0.46 (0.21-1.01)	0.053	
Ann Arbor Stage at Diagnosis		0.391	
1 vs. 0	1.25 (0.16-9.95)	0.833	
2 vs. 0	0.71 (0.34-1.49)	0.363	
3 vs. 0	2.02 (0.73-5.57)	0.173	
4 vs. 0	0.54 (0.17-1.79)	0.317	
Overdominant Genotype		0.101	0.145
G/G-A/G vs. A/A	0.52 (0.24-1.13)	0.101	
Age in Years*		0.371	
15-19 vs. 0-14	0.57 (0.08-4.25)	0.586	

20-40 vs. 0-14	1.06 (0.48-2.32)	0.892	
41-55 vs. 0-14	0.46 (0.21-1.01)	0.054	
Ann Arbor Stage at Diagnosis		0.585	
1 vs. 0	1.77 (0.21-15.28)	0.604	
2 vs. 0	0.74 (0.36-1.54)	0.423	
3 vs. 0	1.58 (0.57-4.37)	0.382	
4 vs. 0	0.54 (0.16-1.78)	0.310	
